# Single-shot dynamics of dual-comb generation in a polarization-multiplexing fiber laser

**DOI:** 10.1038/s41598-023-46999-9

**Published:** 2023-11-11

**Authors:** Alberto Rodriguez Cuevas, Igor Kudelin, Hani Kbashi, Sergey Sergeyev

**Affiliations:** 1 College of Engineering and Physical Sciences, Aston Institute of Photonic Technologies, Aston University, Birmingham, B4 7ET UK; 2 National Institute of Standards and Technology, 325 Broadway, Boulder, CO 80305 USA; 3 Department of Physics, University of Colorado Boulder, 440 UCB, Boulder, CO 80309 USA

**Keywords:** Optical physics, Optical techniques

## Abstract

Dual optical frequency combs have been a recurrent case of study over the last decade due to their wide use in a variety of metrology applications. Utilizing a single cavity laser to generate a dual comb reduces system complexity and facilitates suppression of common noise. However, a dual-comb regime in single cavity lasers tends to be more unstable and difficult to achieve. Therefore, having a better understanding about the way they are generated could improve and automate their generation and control. In this paper, we investigate the build-up dynamics and collision of dual comb in a polarization-multiplexing ring-cavity fiber laser using DFT (Dispersive Fourier Transform) method. We observe a bunch of meta-stable short-lived mode-locking states before the laser entered the dual-comb mode-locking state. The energy level of this short-lived initial pulses determines its evolution. If it decreases too much, the pulse will eventually collapse while if it stays above certain level, it will be successfully generated. The results presented in this paper increase the understanding of dual-comb generation inside a single cavity laser and may contribute in future attempts to increase the stabilization of this regime.

## Introduction

An optical frequency comb (OFC) is an array of uniformly distributed optical frequencies. In the time domain it is a series of ultrashort pulses with nearly identical repetition rate. OFC have been used since the beginning of the twenty first century^1, 2^. Due to its high precision in the time and frequency domains its applications are diverse^2–6^. Certain applications require two frequency combs operating simultaneously and with a known frequency difference between them. These applications include: spectroscopy^5^, LIDAR^6^ or optical communications^7^ among others. There are various ways of frequency comb generation: mode-locked lasers, electro optical modulators and four-wave parametric generation. A significant scientific trend in dual-comb generation within fiber cavities is the adoption of single-cavity configurations^8^. Unlike the approach using stabilization and synchronization of two OFCs, the offset between the two combs (differences in carrier envelope offset frequencies and repletion rates) shows superior long-term stability caused by mutual coherence between the pulse trains in a single cavity. Thus, in the context of applications, the absence of the stabilization techniques makes a single cavity-based dual-comb a cost-effective solution^8^. Within this approach, there are several subgroups or techniques that can be employed, including wavelength frequency multiplexing, polarization multiplexing, cavity multiplexing, circulation direction multiplexing, and extra-cavity methods^8^. Each of these techniques offers its own unique benefits and considerations for generating dual combs. All these methods differ in the structure of the cavity introducing a component or a group of components that eventually create two trains of pulses with slightly different repetition rates. These pulses are usually solitons^9^, and they are able to maintain the shape of its waveform over time while propagating due to its balance between dispersion and non-linearity. They can also merge and form soliton molecular complex, maintain its integrity even in the event of collision with other solitons^10, 11^. In fact, a dual-comb system generates two trains of solitons travelling with a slightly different speed within the cavity. The study of the dynamics of these solitons has been done by several authors^10, 12–17^. Given the variable separation of two combs ranging from hundreds of femtoseconds to tenths of nanoseconds, the oscilloscope’s GHz bandwidth can’t provide real-time observation of the dual-comb build-up dynamics. To address this issue, the Dispersive Fourier Transform (DFT) is widely in use^18–27^. This method stands as the best option given the non-repetitive nature of the events that occur in ultra-short times when the dual-comb regimes are generated. DFT works on the principle of chromatic dispersion. When a pulse of light passes through a dispersive media, for example standard telecommunication fiber, the pulse broadens due to both the difference in propagation velocity of each wavelength and the different interaction of each wavelength with the transmission media. In normal dispersion media, the longest wavelengths will generally travel faster than shorter wavelengths, contributing to the broadening of the pulse. Eventually, each pulse is mapped to a temporal waveform that is analogue of the far-field (Fraunhofer) diffraction pattern in the spatial domain^17^. The temporal resolution of the DFT is limited by the sampling rate of the oscilloscope. The spectra resolution of the DFT measurements, limited by the bandwidth of the used equipment, can be estimated as^17^:

1 $$\Delta \lambda =1/\left(\Delta f\cdot D\cdot z\right)$$

Where ∆f the oscilloscope sampling frequency, D is the group velocity dispersion, and z is the length of the dispersive media (for example, standard telecom fiber).

Previous studies of the dual OFC build-up dynamics using DFT reveal different stages of dual comb evolution, central wavelength shifts in opposite directions and vector-soliton collisions^18, 25^. Unlike the previous studies, herein, for the first time, we analyze the successful and unsuccessful dual-comb formation from spikes generated in the background noise, and the build-up dual-comb regime from a single-comb regime. Collision dynamics of the solitons in the same dual-comb regime is also reported*.* Understanding how the dual comb is generated inside the cavity can give us insights for optimal ways of generating dual comb with increased performance and optimize the relative long-term stability between each comb^23^. Eventually being able to fully characterize solitons or ultrashort pulses and having a reliable frequency conversion mechanism give way to an extensive number of applications: multi-photon microscopy, Raman spectroscopy, supercontinuum generation, frequency comb-based measurements or parametric-based two-photon interference among others^24^.

## Experimental setup

**Figure 1:**
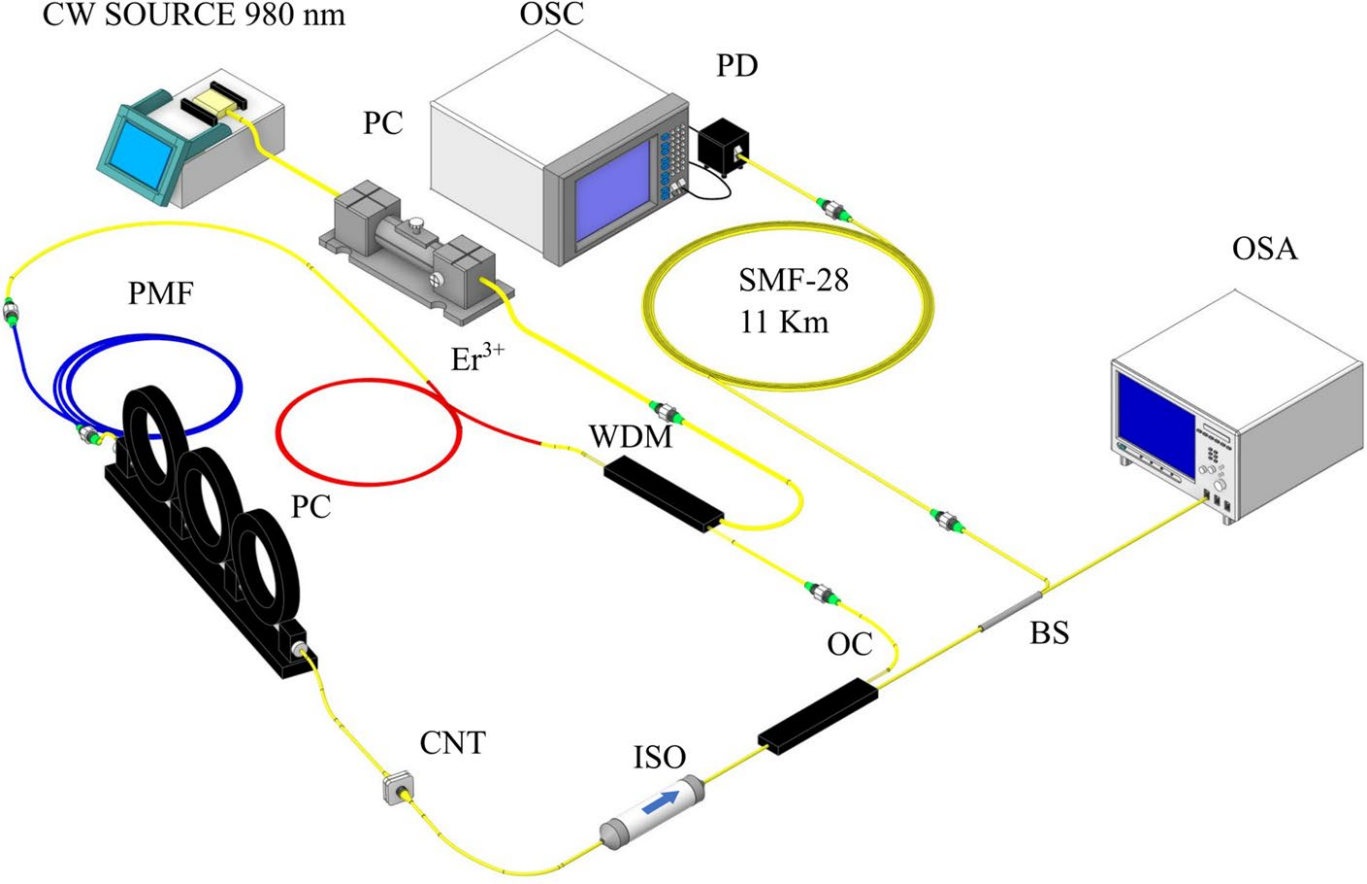
Representation of the laser cavity consisting of the following parts: isolator (ISO), optical coupler 90–10 (OC), wavelength-division multiplexing (WDM), polarization controller (PC), carbon nanotube (CNT), 1 m of polarization maintaining fiber (PMF), 0.8 m of Erbium-doped fiber (Er^3+^), and a continuous wave laser pumping at 980 nm (CW SOURCE). In combination with a 1 × 2 beam splitter (BS) an optical spectrum analyzer (OSA), and a 11 km-long SMF-28 segment connected to a photodiode (PD) that is connected to an oscilloscope (OSC).

The setup we use in this work is a polarization-multiplexed dual-comb laser, consisting of a ring cavity that is mode-locked by home-made carbon nanotubes^28^. Figure 1 displays a schematic representation of the fiber cavity. A 0.85-m-long Er-doped fiber (Liekki Er80-8/125), pumped using a 980 nm laser diode through a 980/1550 nm wavelength division multiplexer, has been used as an active gain media. A 1-m-long polarization-maintaining fiber (PM fiber) has been used inside the cavity to generate polarization multiplexing mode-locked regime. This polarization multiplexing, responsible for dual-comb generation, is achieved by adjusting the polarization states inside the cavity with a polarization controller, placed after the PM fiber. A 51 dB dual-stage polarization-independent optical isolator is inserted in the laser cavity to produce unidirectional lasing. Finally, an optical coupler closes the loop maintaining 90% of the intensity inside and re-directing 10% outside. The total length of the cavity is 16 m, corresponding to the fundamental repetition rate of 12.657 MHz (79 ns) with a repetition rate difference between each comb of around 255 Hz. The detection and measuring systems include a 50 GHz dual-window photodetector (XPDV2320R, II-IV) connected to a 33 GHz high performance sampling oscilloscope (Agilent DSOX93204A Infiniium).

In our previous publication^28^, we conducted a comprehensive experimental characterization of the dual-comb generation. To achieve this, we utilized PM fiber and tunable in-cavity polarization controller. Through this setup, we successfully generated dual combs with frequency differences ranging from 5 to 255 Hz. Notably, we achieved particularly stable combs within the frequency range of 110–250 Hz^28^. For instance, we observed that the dual-comb regime with a frequency difference of 255 Hz remained stable, exhibiting only a slight drift of 6 Hz over a 6-h period (equivalent to 15 mHz in 60 s).

In order to separate the combs, we utilized the output polarization controller and a polarization beam splitter (PBS). This configuration resulted in an extinction ratio of 16.5 dBm for the two combs, effectively distinguishing and characterizing their individual properties.

In order to elucidate the build-up dynamics concerning stable and unstable dual-comb generation, we employed DFT with an oscilloscope with a sampling frequency of 33 GHz. The experimental setup involved the use of an 11 km-long standard telecom fiber with a group dispersion velocity of 17.4 ps nm^–1^ km^–1^. By solving Eq. (1), we achieved a spectral resolution of 0.158 nm.

To obtain the necessary data, we meticulously adjusted the polarization controller to establish a stable dual-comb generation regime. Subsequently, the trigger of the oscilloscope was set to a level where overlapped pulses would activate it. Then, the pump laser was switched off and on within a brief period of time. The newly generated combs triggered the oscilloscope, allowing us to record a 12.7 ms (∼321,500 round trips) trace both before and after the trigger activation. This provided valuable insights into the pulse onset dynamics, from initial noise to stable dual-comb generation.

To investigate the build-up dynamics from a single comb to a dual-comb, we employed a similar approach. Instead of switching the laser on and off, we strategically moved the polarization controller to a position where the dual-comb generation occurs. Subsequently, we slightly adjusted the polarization controller to transition back to a single comb regime. The trigger was then set, and the polarization controller was rotated back to its original position. Through this meticulous setup, we accurately recorded the dual-comb formation, as the trigger activated only when both pulses perfectly overlapped with each other.

## Results and discussion

Herein we present the results obtained by DFT of successful and unsuccessful build-up, single comb to dual comb transition and propagation dynamics. These results provide observational evidence of the dynamics that lead to the generation of two optical frequency combs inside a single cavity using polarization multiplexing as the method to generate them.

**Figure 2:**
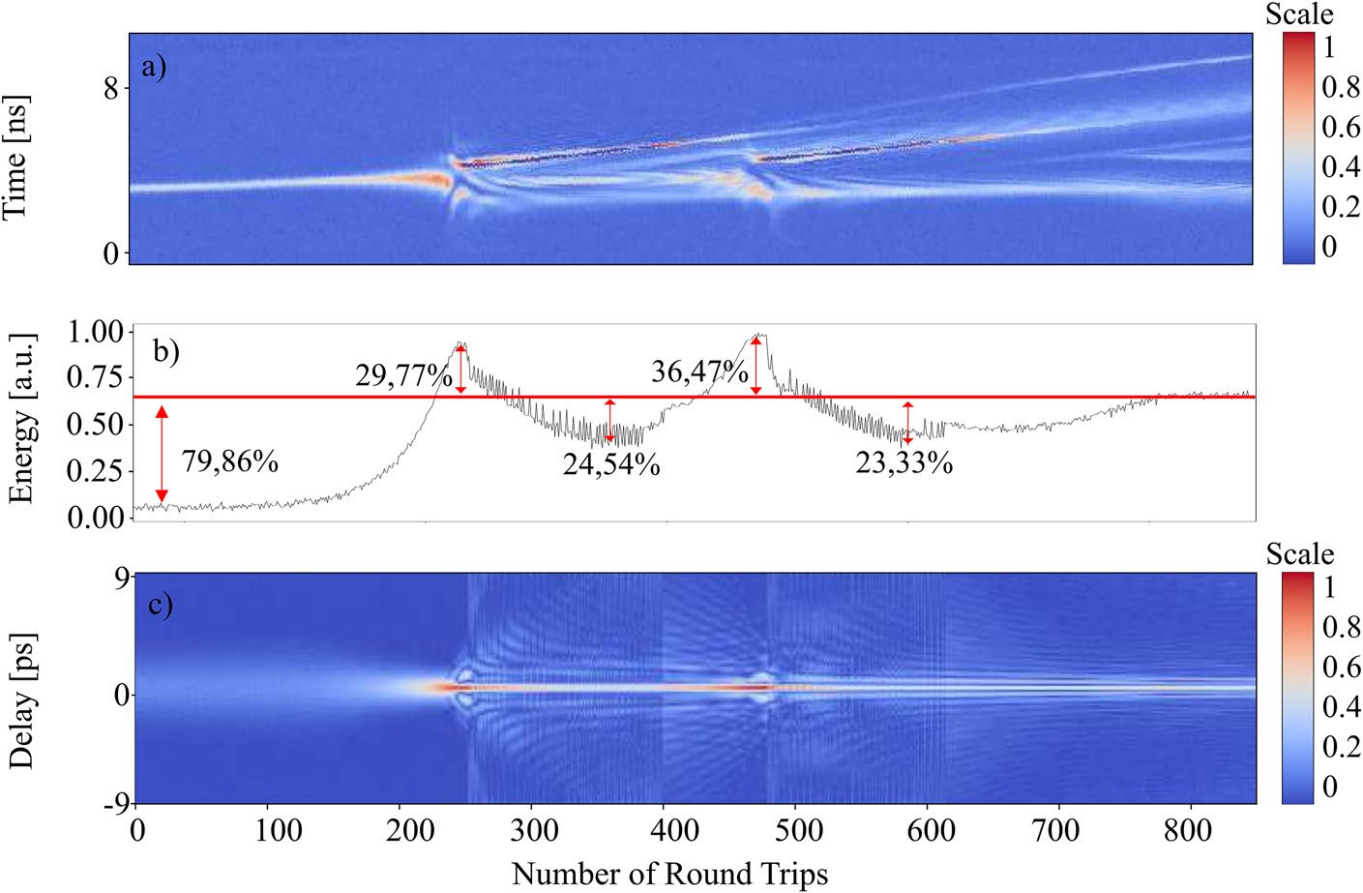
The build-up dynamic of the dual comb. (**A**) Intensity dynamics obtained from the oscilloscope. (**B**) The corresponding pulse energy. (**C**) First order autocorrelation trace.

**Figure 3:**
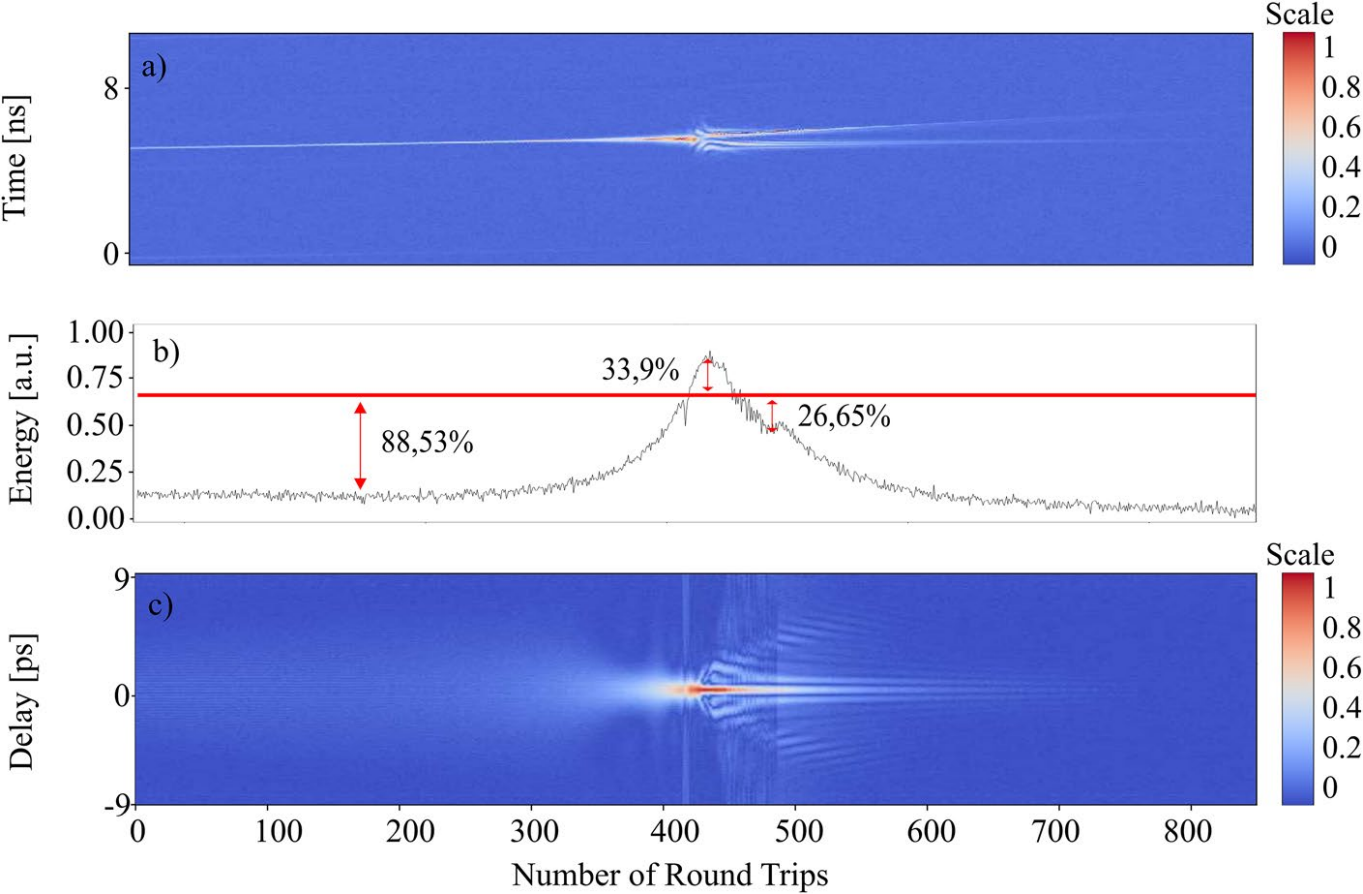
The build-up dynamic of the dual comb for one of the spikes that collapses before the dual-comb formation. (**A**) Intensity dynamics obtained from the oscilloscope. (**B**) The corresponding pulse energy. (**C**) First order autocorrelation trace.

The dynamics of dual-comb build-up are presented in the Fig. 2. As mentioned before they are recorded by switch the pump power off and on again starting from an initial dual-comb regime. We can see that initially, in the system, there is just background noise, but after several thousand round trips, and due to the effect of the nonlinear absorbent, some ultrashort spikes start to become dominant over the rest of the noise. Most of them collapse but some of them grow to a point where they experience spectral broadening. Eventually, they split into two independent and stable pulses. In this build-up process we can observe that previous spikes get wider and split into two pulses but eventually collapse short after, see Fig. 3. The observation in Fig. 3 is not unique. Several energy spikes repeat the same process immediately after each other until one of them successfully separates into two. Once the two pulses start propagating along the cavity, the carbon nanotube (acting as the mode-looking instrument) filters out all the remaining noise and prevents the generation of more energy spikes.

The whole process of dual-comb generation from the point where the narrow spike appears took approximately 3500 round trips (276 µs) and it occurs in two steps. The time between each step took roughly 227 round trips or (17.93 µs). This two-steps generation can be clearly identified in the pulse energy graph (Fig. 2b), where the second energy increase is slightly higher and wider than the first one. After the second intensity peak the overall intensity tends to get stable over time in correlation with the stability of propagation of both combs along the fiber ring cavity. We consider the stable energy value of the dual-comb propagation as the reference value in the Figs. 2 and 3. The initial spike that triggers the formation of the dual-comb experiences a steady increase in energy. At a certain point, when this energy reaches approximately 20.14% of the final energy, the spike’s energy undergoes an exponential increase. It surpasses the final energy by around one third and subsequently decreases to a minimum level, which is approximately 24.54% lower than the reference value. This pattern repeats, with the spike’s energy peaking at approximately 36.47% above the reference value before decreasing to a valley around 23.33% below it. Following this second valley, there is a gradual increase in energy until eventually reaching a stable regime, allowing the two solitons to propagate independently. The pulse energy refers to the sum of all the energy in the laser cavity during one round trip. This includes not only the energy in the laser pulse, but also any noise or fluctuations that may be present in the cavity. To calculate it we divide the original 12.7 ms-long OSC trace in segments of 79.004968 ns (round trip time of the slow pulse). Then we sum the dB of each point inside these segments and plot it with the corresponding time. The obtention of the Auto-Correlation Function (ACF) consists of iterating through each row of the original DFT data and performing the inverse Fourier transform in each of them. The absolute values of each iteration are appended to the new matrix and represented in the figures.

In Fig. 3, unlike on Fig. 2**,** the separation of the spike into two pulses collapses and the dual-comb generation is unsatisfactory. Indeed, the energy evolution can provide us with additional information about that process. After the first energy increase the energy decay quickly and eventually collapses into the background noise. The collapse of this initial spike is immediately followed by the formation of the dual comb that is shown in the Fig. 2, indeed the spike that collapsed and the spike that succeed coexisted in competence just until one of them succeeded becoming an actual dual comb travelling along the cavity. Nonetheless, the pulse energy shows us that the first part of the build-up dynamics is roughly the same with an exponential increase in the spike’s energy from 10% of the reference value to 33.9% and then to a valley 26.65% below. However, once the energy of the spike gets to this point it fails to generate a second peak and its energy starts to decrease until the peak eventually vanishes. In short, there is an energy trigger that need to be surpassed to achieve dual-comb generation. The significance of energy dynamics during build-up is similar to dynamics in the other dual-comb systems^13^. Videos of the build-up dynamics can be found in the supplementary materials (see Supplementary Video V1).

A successful dual-comb build up produces a frequency difference, measured in the RF spectrum analyzer, of 255 Hz equivalent to a difference in pulse delay of 1407 ps per round trip. The round-trip time of the slow axes is 79.004968 ns while the round-trip time of the fast axes is 79.003561 ns. These round-trip times must be evaluated considering that the cavity length of the laser is 16.363 m. Therefore, the refractive index difference is 2.573*10^–5^, and the beat length of the cavity is 0.0605 m. Eventually, we obtain a difference in propagation between both pulses of 103.925 m^−1^ and a phase delay of 1700.52 oscillations.

**Figure 4:**
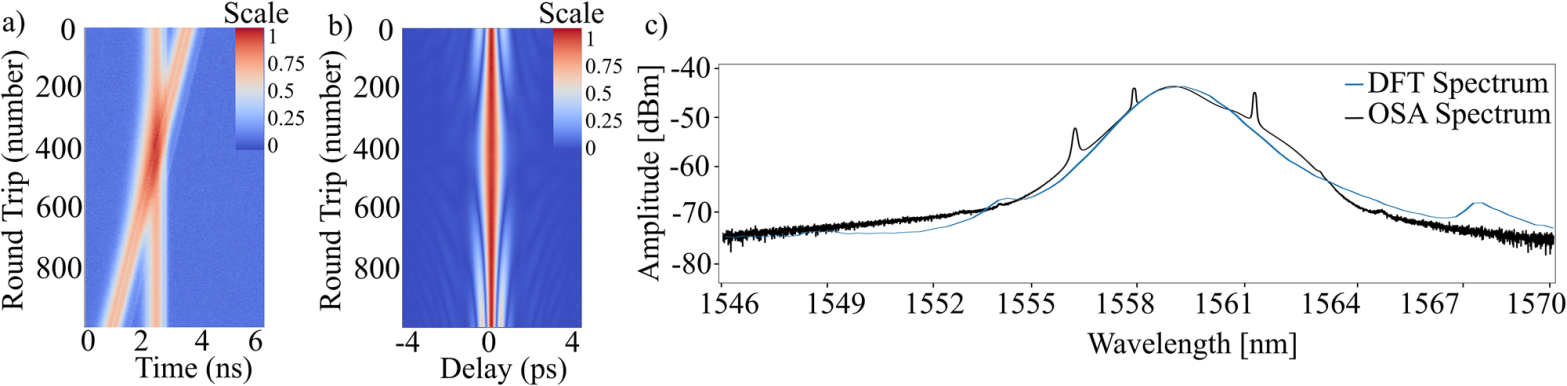
The interaction of the dual comb at the moment of collision. (**A**) DFT trace of the collision. (**B**) Autocorrelator trace of the collision. (**C**) Optical spectrum of the light pulses obtained with the OSA in black and with the DFT in blue.

When it comes to the collision of the solitons within the cavity once the dual-comb regime has been achieved the collision does not affect the shape of either soliton, as shown in Fig. 4a and b. However, we have observed a case where a soliton molecule composed of multiple solitons can lose one of them after the interaction. Nonetheless, among the multiple observations of collision that have been captured with DFT there is not a single case where the intensity lines change the trajectory. Indicating that the collision does not alter the propagation velocity of the pulses in any way. Pulse collision information is highly relevant from an application perspective since some applications based on dual-comb such as dual-comb lidar required from two independent combs that remain stable while they are colliding with each other^6^. This pulse behavior can be observed in the autocorrelation traces of the Fig. 4b. Further observations of soliton molecules reveal a more instable behavior. Videos of the soliton collision, soliton molecule collision are included in supplementary materials within this publication (see Supplementary Videos V2, V3 and V4).

In addition to the build-up and propagation dynamics DFT can reveal important short-term spectral dynamics. These dynamics are important because soliton collisions could induce short-term instabilities which might affect the measurements^26^. Figure 4c offers a comparison of the optical spectrum of the pulses derived from the DFT and the optical spectrum captured with the OSA. When the optical spectrum is obtained with the DFT it must be averaged over a number of round trips, in our case 300. These round trips are acquired when the dual-comb regime is stable. The time to wavelength correspondence in the DFT intensity dynamics map is 1 ns equivalent to 5.214 nm. However, when applying these conversions to the figures that represent time vs DFT it must be taken into account that this conversion can only be applied directly when we have a section of the map with a single soliton, in the case there are more than one propagating at different velocities it can lead to a mistake since this time-to-wavelength conversion can only be applied to one of them*.*

**Figure 5:**
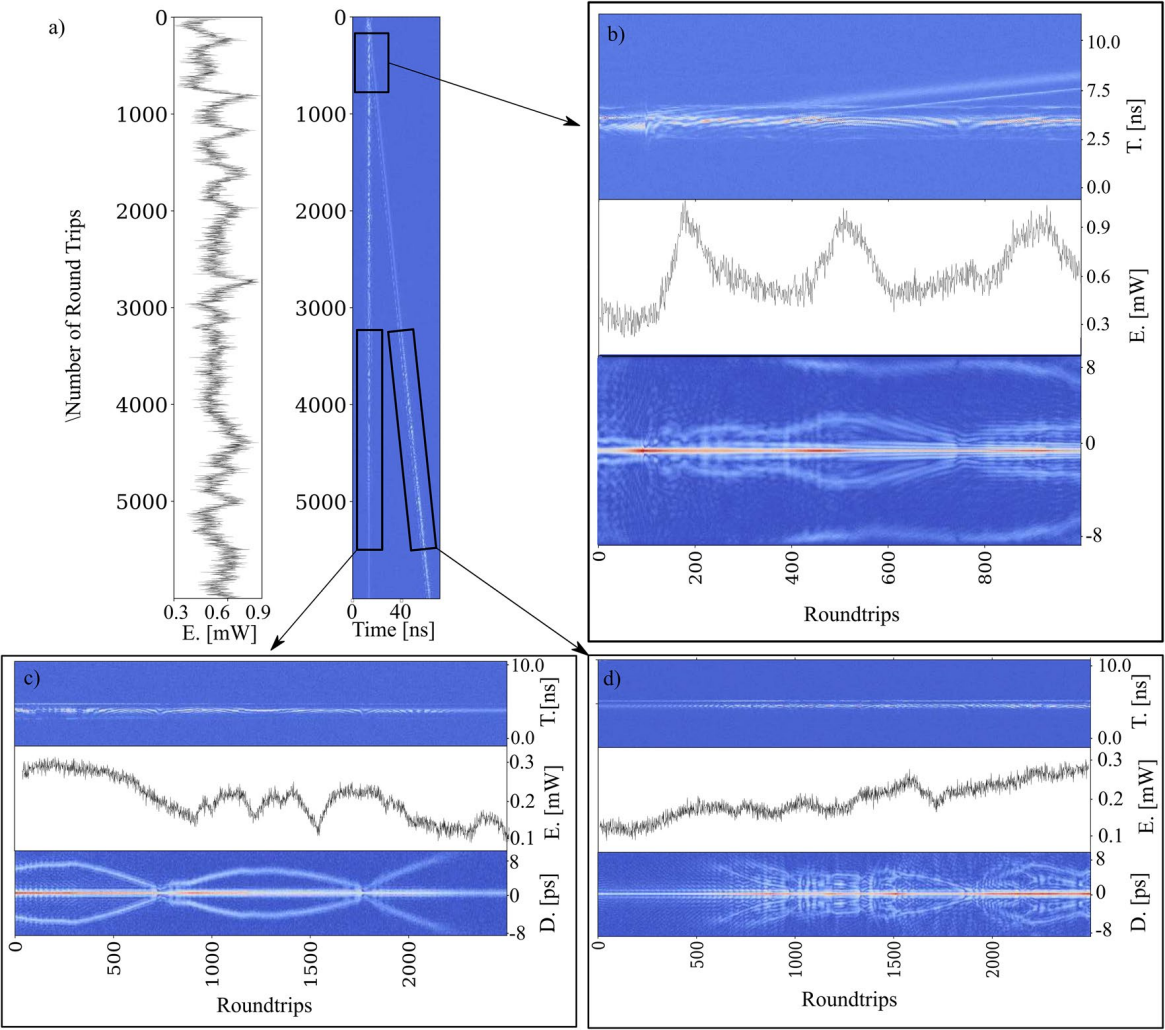
Dual-comb generation from a single comb regime. (**a**) Dual-comb generation obtained with DFT and its corresponding pulse energy. (**b**) Zoom-in of the actual dual-comb generation section, pulse energy and autocorrelator trace. (**c**) Zoom-in of the slow axes section where there is an energy transition, its pulse energy and its autocorrelator trace. (**d**) Zoom-in of the fast axes section where there is an energy transition, its pulse energy and its autocorrelator trace.

Another important dynamic occurs when the dual comb is generated from a single comb. In this last case a new comb splits off from the original one, without altering the speed of propagation of the original one. Having a mode-locked regime with a single pulse per round trip we can adjust the polarization controller inside the cavity to a position where two different trains of pulses start to circulate along the cavity. These pulses circulate at a slightly difference in repetition frequency due to the polarization-multiplexing nature of the fiber cavity. Figure 5 presents the build-up dynamics for this case. The AC traces obtained by calculating the Fourier transform of the intensity map show a pulse pattern that evolves in a way that the slow axis losses its multi-pulsing after some round trips while the fast axe develops a multi-pulsing pattern almost simultaneously. Figure 5a and more closely Fig. 5b, evidence some differences respect to the build-up dynamics shown in the Fig. 3. On the one hand the 1-to-2 transition is more chaotic than the build-up and the energy fluctuates for a longer period of time. On the other hand, there is a pulse energy transition from the slow to the fast axes after roughly 3000 round trips from its creation. The main reason for these two phenomenon is the adjustment in the intracavity polarization produced when the polarization controller is moved during the process. While in the case of the build-up the polarization controller remains always in the same position. This difference can explain the succession of peaks and valleys presented in the energy level right before and after the separation into two combs and evidences the need for high precision electronically-driven polarization controller. The original pulse (single-comb) becomes highly unstable in the round trips previous to its separation. Its pulse energy shows a noisy behavior while the AC trace indicates complex multi-pulsing patterns. Once the separation has occurred and for the first 1000 round trips there are several consecutive peaks and valleys of energy. Another difference seeing in the Fig. 5c and d is the energy stability and pulse transition between each comb. The slow comb comes out of the separation with a noisy behavior while it transits to a more stable and less energetic state. Simultaneously, the fast comb experiences the opposite transition, increasing its overall energy and the autocorrelator trace shows an evolution from a single pulse towards a complex and unstable pulse distribution. This energy transfer from one comb to another can be explained by the change in the polarization controller and therefore in the birefringence that affects the overall pulse propagation.

Propagation and build-up dynamics are conditioned by the stability of the energy levels in every round trip. Internal variations in the energy level are present in both generation and collapse of the dual-comb and therefore are intrinsically related with the stability of the regime. Therefore, an essential consideration is that any external perturbation that influences the energy level inside the cavity such as vibrations, changes in the environmental conditions such as temperature or pump power instability can trigger the collapse of the dual-comb regime. To investigate this effect, we recorded several DFT maps every 10 min of a stable dual-comb regime that lasted for an hour, as well as in vivo observations. Over the first 56 min, the regime was stable, and the intensity dynamics of the DFT maps and OSA spectrum showed no differences, except for changes in the propagation speed resulting in differences in the round-trip time of 5–10 fs per hour, or 0.8 to 1.6 Hz in repetition rate frequency. However, in the last 4 min, increased instability was observed, with clear variations in the light spectrum composition between successive samples obtained every 2 s. The more frequent and intense these variations were, the closer the dual-comb regime was to collapsing into a single-comb regime.

These instabilities in the spectrum composition can be seen as an early warning signal and can prevent the collapse of the regime by introducing corrective measures that extend the lifespan of the dual-comb regime. It should be noted that the authors of this research consistently observed the pattern of increased instability leading to dual-comb collapse in previous works^28^. This observation opens the door to evaluate the influence of programed adjustments in the system’s power and internal polarization in an effort to ease the dual-comb generation once the birefringence of the cavity has been adjusted to the adequate levels.

## Conclusions

In this study, we have demonstrated the spectral and temporal dynamics of dual-comb formation within a fiber ring-cavity laser, from a mode-locked regime and from power off to the establishment of a stable dual-comb propagation along the cavity. Unlike the previous study of the dual-comb build-up dynamics^18, 25^, our findings reveals the mechanism of successful and unsuccessful dual-comb formation from spikes generated in the background noise. The pulse energy of the initial short- lived mode-locked states plays a critical role in the build-up dynamics. If the energy level decreases under a certain level it tends to collapse, while if it remains over a certain value there is a successful separation into two orthogonally polarized independent pulses. Thus, the experimental observations pave the way to understanding the formation a dual-comb regime in the same cavity in the context of development of industrial-grade dual-comb laser sources. Moreover, the tunable dual comb can be developed by using the output autocorrelation trace resulting from DFT data combined with machine learning based evolution algorithm and electronically-driven polarization controller^27^.

### Supplementary Information

Supplementary Video 1.

Supplementary Video 2.

Supplementary Video 3.

Supplementary Video 4.

## Data Availability

The datasets used and/or analyzed during the current study available from the corresponding author upon reasonable request.
